# A case study on developing students' problem-solving skills through interdisciplinary thematic learning

**DOI:** 10.3389/fpsyg.2025.1447089

**Published:** 2025-05-26

**Authors:** Chuntong Zhang, Pei Wang, Xinwu Zeng, Xiaohong Wang

**Affiliations:** ^1^College of Education, Shanghai Normal University, Shanghai, China; ^2^Wuhuang Middle School, Ziyang, Sichuan, China; ^3^Chengdu Mianshi Foreign Language School, Chengdu, Sichuan, China

**Keywords:** interdisciplinary thematic learning, problem-solving skills, middle school students, case study, three-dimensional support

## Abstract

Interdisciplinary thematic learning, driven by thematic tasks and real-world problems, is an effective vehicle for cultivating students' problem-solving skills and individual development. However, traditional subject-based teaching has long compartmentalized knowledge into discrete academic disciplines, diverging from the nature of real-world problems and their solutions. Although interdisciplinary thematic learning aims at developing students' key abilities for future life, few empirical studies have examined whether interdisciplinary thematic learning has developed Chinese students' problem-solving skills during the compulsory education phase. Moreover, interdisciplinary thematic learning fully develops and makes use of school subject resource, which plays an important role in developing students' problem-solving ability, but ignores the influence of student foundation and social environment and other factors. This study investigates the development of problem-solving skills among 47 middle school students from a compulsory education institution in Sichuan Province mainly adopts interview, observation and case study. The findings indicated that the process of interdisciplinary thematic learning includes three-dimensional support composed of students' foundation, subject resources and social environment, which jointly support the development of interdisciplinary thematic learning, thus realizing the development of students' problem-solving ability. In the future, while continuing the advantages of multi-case studies, we should jointly carry out single-case and quasi-experimental design study to explore the depth of the study. In addition, it is necessary to consider teacher guidance, digital technology and computing thinking skills when choosing research perspectives in the future. From this, more conclusive and comprehensive conclusions and suggestions are put forward.

## 1 Introduction

Problem-solving ability is key to individual growth and success and a crucial driver of societal progress and development. The Organization for Economic Co-operation and Development (OECD) identified the ability to solve complex problems as one of the core competencies (OECD, 2005), and a critical skill for future employment (Ananiadou and Claro, 2009). However, traditional subject-based teaching has long compartmentalized knowledge into discrete academic disciplines, diverging from the nature of real-world problems and their solutions. This approach has led to a disconnect between school-taught knowledge and its flexible application to complex real-world issues, creating a divide between school education and the real world. The traditional teaching process, dominated by this logic, does not facilitate the development of students' problem-solving abilities. Even though problem-solving activities are highly valued in subject teaching (Heyworth, 1999), traditional subject-based instruction often isolates the development of problem-solving skills within individual disciplinary boundaries. Each discipline independently applies its distinct knowledge and skills to explore solutions (Klein, 2017), resulting in different solutions to the same problem from various disciplinary perspectives. This fragmentation makes it difficult to form a comprehensive system for problem-solving, leading to incomplete problem-solving abilities in students that hinder their ability to tackle complex problems in the future and societal innovation and progress. Consequently, numerous studies have begun to explore the development of students' problem-solving skills from an interdisciplinary perspective (AAAS, 2011; NAE and NRC, 2014; NRC, 2005).

To address the fragility and uncertainty of future global development, United Nations Educational, Scientific and Cultural Organization states that curricula should focus on interdisciplinary learning to support students in acquiring and creating knowledge and developing students' key competencies in applying knowledge to problem-solving (UNESCO, 2021). Interdisciplinary thematic learning is an activity-driven curriculum focused on thematic tasks and real-world problems. It breaks down the barriers between school learning content and the real world and bridges the gaps between different school subjects and between school and the outside world. Interdisciplinary knowledge is increasingly vital for understanding and addressing complex real-world issues and problem-solving ability is essential to the successful implementation of interdisciplinary thematic learning (Herde et al., 2016; OECD, 2018). Therefore, there is a close connection between interdisciplinary thematic learning and the development of students' problem-solving abilities.

The 2022 “Compulsory Education Curriculum Plan” by the Ministry of Education of the People's Republic of China, explicitly emphasizes the need to enhance the integration of within-subjects knowledge and to design interdisciplinary thematic teaching systematically. This approach aims to develop students' abilities to apply knowledge comprehensively in real-life situations (Ministry of Education of the People's Republic of China, 2022). The curriculum standards for each subject support the implementation of interdisciplinary thematic teaching based on their essential characteristics and offer specific interdisciplinary teaching cases that highlight the unique features of each subject. These standards advocate that interdisciplinary thematic learning activities should focus on the core goal of developing students' problem-solving abilities by breaking through artificial boundaries within and between disciplines. This shift moves away from the traditional “subject-centered” discrete teaching model to a new model that integrates disciplines and fosters dynamic development centering on the development of students' abilities.

By implementing the Compulsory Education Curriculum Plan and the curriculum standards for various subjects, the Chinese education sector has been promoting interdisciplinary thematic learning at the compulsory education level for over 2 years. Chinese researchers and practitioners are in the ongoing process of exploring this approach. The focus of their research involves supplementing basic standards and norms from different perspectives and collaboratively addressing issues that arise in practice. This ensures that interdisciplinary thematic learning activities stay on the right track in achieving their overall objectives. The significant achievements in interdisciplinary thematic learning in the Chinese education field are evident in the widespread awareness of its value orientation and a general understanding of its conceptual ideas for practical implementation (Wu, 2022; Hong and Xiao, 2023; Li, 2023; Wu and Tian, 2023a,b). However, it is also essential to recognize areas for improvement. Despite the requirement for each subject to devote no < 10% of class hours to interdisciplinary thematic learning, few empirical studies have examined whether and how these activities develop students' problem-solving abilities. Therefore, this study aims to explore the following research questions from a practical perspective:

1. What impact does interdisciplinary thematic learning have on middle school students' problem-solving abilities?
2. How does interdisciplinary thematic learning influence middle school students' problem-solving abilities?

To address these research questions, this study will review and analyze the literature on the following aspects: the nature of interdisciplinary thematic learning and problem-solving ability; the theoretical foundations of the cognitive process of problem-solving; and the logical pathways through which interdisciplinary thematic learning facilitates the development of problem-solving skills. Through examining these four areas, this study will formulate research question/s and hypotheses related to the research question/s.

## 2 Literature review

### 2.1 Interdisciplinary thematic learning

Interdisciplinary thematic learning evolved from subject-based thematic learning. It is a form of integrated learning that utilizes themes to create an authentic, holistic, and meaningful learning environment (Assahary et al., 2017; Ain and Rahutami, 2018). It consolidates the specialized concepts, learning materials, and knowledge branches of a particular subject under specific themes (Syamsuddin et al., 2021). Providing students with engaging, enjoyable, and valuable learning opportunities (Min et al., 2012), adds a depth of meaning to the learning process. Interdisciplinary thematic learning builds on this foundation by adding the element “interdisciplinary.” To understand interdisciplinary and the concept of “interdisciplinary thematic learning,” it is essential to grasp the meaning of the character “跨.” In *Shuo Wen Jie Zi*, “跨” is explained as “to cross” or “to traverse” (Xu, 2018). Additionally, in the *Xunzi* · *Confucian Effect*, “跨” is used to convey the idea of crossing boundaries. “Therefore, the outer gate does not need to be closed, and one can walk across the world without borders” (qí, derived from “圻,” means boundary; Xun, 2015). This interpretation highlights “跨” as crossing boundaries. Combining the connotation of subject-based thematic learning and the ancient interpretation of “跨,” interdisciplinary thematic learning points to breaking the independent states within and between subjects and promoting a fusion and integration of disciplines around specific themes. This approach facilitates the convergence of various subjects into a coherent and integrated learning experience under defined themes.

In the early twentieth century, Professor Woodworth of Columbia University in the United States coined the term “interdisciplinary” as a proprietary noun, emphasizing the breaking down of barriers between knowledge disciplines and gradually evolving toward a comprehensive knowledge system. After nearly 100 years of iterative updates, the concept of interdisciplinary is once again a subject of research in the twenty-first century. A key reason is that since the beginning of the century, major issues in fields such as society, politics, economy, environment, and technology are highly complex and interrelated. Thus, relying on any single discipline, profession, or skill makes it difficult to form a complete system of solutions. Instead, interdisciplinary knowledge, theories, and methods are required to guide problem-solving (Yuan, 2020). In response to the new requirements of the times, this research topic has attracted the attention of many researchers (Kidron and Kali, 2015). Thompson-Klein's classification refers to “breaking the boundaries between disciplines” as interdisciplinary, rather than multidisciplinary. Interdisciplinary integrates knowledge from different disciplinary fields to create new integrated knowledge whereas multidisciplinary combines the characteristics of various disciplines weakening the interactions between disciplines to maintain their characteristics (Thompson-Klein, 2010). The school curriculum is the consolidation of different disciplines within the school with reasonable arrangement and organization to carry out class-based and graded education (Cui and Guo, 2023). Interdisciplinary thematic learning belongs to the category of the school curriculum. It focuses on practical exploration and problem-solving under a specific learning theme to achieve the integration of different disciplinary contents and opens up new paths for curriculum integration.

### 2.2 Problem-solving ability

Problem-solving involves a series of goal-directed cognitive operations when a solution is unclear. Individuals transform the current state into a goal state through behavior (Anderson, 1980; Mayer, 2003). Problem-solving ability refers to the capacity of individuals to engage in cognitive processing to understand and resolve problem situations when there is no clear solution (Liu et al., 2022). It encompasses the individual's capacity to select, construct, or activate a solution and monitor the implementation process, thus reflecting their willingness to engage in problem-solving to realize their potential as creative and reflective citizens (OECD, 2013). At its core, problem-solving ability involves the cognitive process of transforming a known situation into a goal situation (Chen and Liu, 2019). From a cognitive perspective, one of the most influential models explaining the human problem-solving process is the problem-solving model proposed by Gick (1986), which is based on research on problem-solving strategies. This model comprises four stages: understanding and representing the problem, seeking solutions, attempting solutions, and evaluating. Initially, problem-solvers need to identify relevant information to determine the nature of the problem. Next, they need to apprehend the meaning of the information to attain an accurate understanding and appropriate representation of the problem. If problem-solvers can evoke a viable solution, an appropriate schema is activated leading to the emergence of a solution. If there is no readily available schema to evoke an immediate response, problem-solvers will design different problem-solving solutions based on the initial and goal states of the problem by comparing, weighing, and selecting from various solutions. Once a solution is selected, the problem-solver enters the trial-and-error stage of executing the plan or attempting a solution. If unsuccessful, adjustments to the plan or changes in the understanding of and approach to the problem are necessary, based on feedback information about the problem-solving outcome. If successful, evaluation of the outcome is required through the search for evidence that confirms or refutes the solution. According to the OECD (2017), the cognitive processing involved in the problem-solving process of a specific task includes exploring and understanding, representing and formulating, planning and executing, and monitoring and reflecting. Exploring and understanding involve establishing mental representations for each piece of information in the problem. Representing and formulating entail constructing graphical, tabular, symbolic, or verbal representations of the problem scenario and formulating hypotheses regarding relevant factors and their relationships. Planning and executing involves setting goals and subgoals to formulate plans and executing the consecutive steps identified in the plan. Monitoring and reflecting involve monitoring progress, responding to feedback, and reflecting on solutions. Assessment of problem-solving ability refers to the value judgment of students' problem-solving abilities based on the collection and analysis of information on the application of knowledge and skills during problem-solving (Wang, 2021). Existing research typically divides problem-solving cognitive processes into stages and sets hierarchical levels, such as levels 0–3 (excellent, good, fair, poor; Szetela and Nicol, 1992; Zhu and Hu, 2021), as criteria for distinguishing different levels of student performance in problem-solving abilities (OECD, 2023).

### 2.3 Information processing theory

Accurately understanding the underlying logic of students' cognitive activities when solving problems requires relying on learning theories that can adequately explain how the human mind processes, retrieves, and stores information. Neuroscientific research indicates that learning involves modifying or adding new synapses in the brain, thereby forming new neural networks and connecting them with existing ones (John et al., 2000). Inspired by computer science, information processing theory regards human learning as an “input-encoding-output” information processing process that entails visualizing the neural connections that occur inside the brain, identifying the activities within the brain's “black box,” and explaining the human learning mechanism. According to the information processing theory, learning is a series of cognitive operations that students engage in among and within the three cognitive architectures of sensory, working, and long-term memory (Raaijmakers and Shiffrin, 1981).

**Figure 1:**
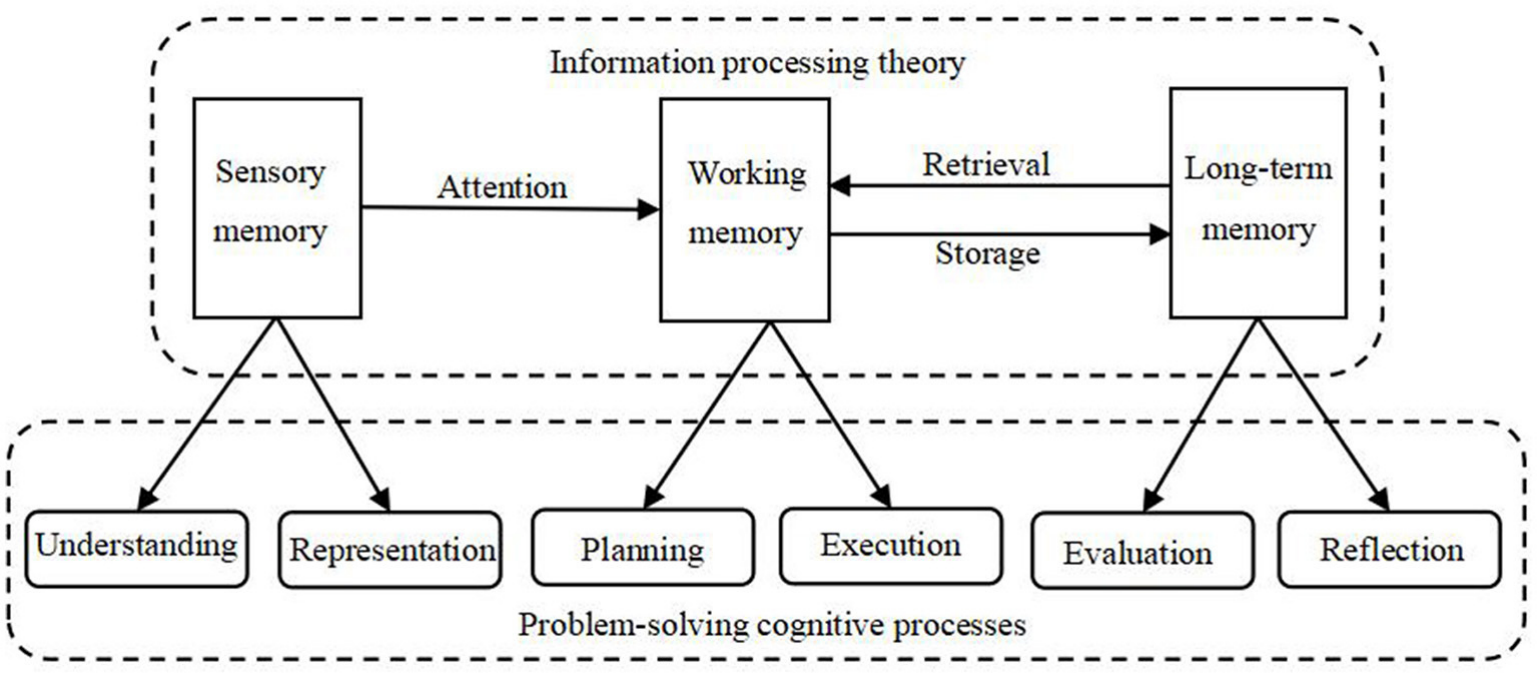
The cognitive process of problem-solving guided by information processing theory.

The psychological processes that students undergo during learning within the framework of information processing theory correspond to the cognitive processes involved in problem-solving, as illustrated in Figure 1. Specifically, when external information is received and registered by the sensory system, students form sensory memory and selectively attend to new information from sensory memory. This process is akin to the psychological processes involved in understanding and identifying problems within a given context. Subsequently, students establish associations between the selectively attended new information from sensory memory and relevant old information retrieved from long-term memory within the cognitive processing space of working memory, thus gaining a meaningful understanding of the new information. This process is similar to the psychological processes involved in proposing and implementing solutions to the identified problems. Finally, students assimilate the newly understood information into existing cognitive structures or restructure existing cognitive structures in long-term memory for storage, facilitating later retrieval and application. This process is akin to the psychological processes involved in evaluating and reflecting on the problem-solving process. Therefore, information processing theory provides theoretical guidance for understanding the cognitive processes that students undergo when solving problems.

### 2.4 Process logic in developing students' problem-solving abilities through interdisciplinary thematic learning

Cross-disciplinary thematic learning is a curriculum activity mode guided by curriculum consciousness and belongs to the category of curriculum. The curriculum involves three factors: students, disciplines, and society (Jin, 2015). Therefore, before the implementation of cross-disciplinary thematic learning, curriculum developers should seek support from three aspects—student foundation, disciplinary resources, and social environment—to promote the development of students' problem-solving abilities. Concerning student foundation, teachers need to understand and focus on the pre-existing conditions of students' development in basic knowledge and skills, learning processes and methods, emotional attitudes, and values (Xhomara, 2020; Creighton and Dewey, 1916; Howe and Berv, 2000), so they can support students' smooth participation in cross-disciplinary tasks and problem-solving. Concerning disciplinary resources, the implementation of cross-disciplinary thematic learning is premised on fully respecting the characteristics of different disciplines (Mansilla and Duraisingh, 2007), integrating resources with unique characteristics from different disciplines (NRC, 2005; Wagner et al., 2011), and enriching solutions to solve problems, thereby deepening the preconceived and executed problem-solving solutions. Concerning the social environment, the implementation of cross-disciplinary thematic learning should establish close connections with the social environment and students' lives (Fischer et al., 2012), mine learning themes, propose real problems, and create practical situations (Brown, 2018; Ruslan et al., 2021; Kirn and Benson, 2018; Ye and Xu, 2023). This will ensure that learning activities contribute educational wisdom and strength to the development of students' abilities to solve complex real-world problems. The implementation of cross-disciplinary thematic learning, integrated with students' foundation, disciplinary resources, and social environment, deepens and expands students' problem-solving processes and provides a platform for exercising and developing students' problem-solving abilities.

### 2.5 Summary

**Figure 2:**
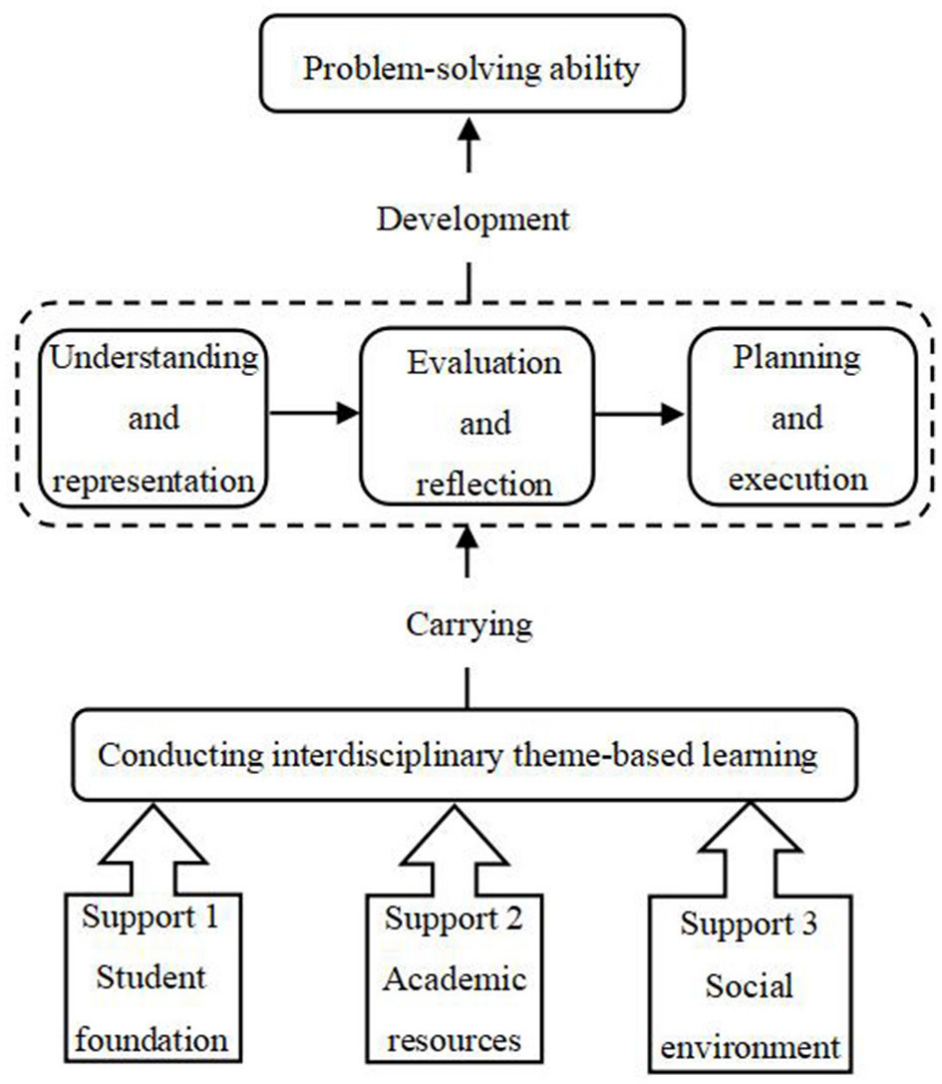
The logical framework for the development of problem-solving abilities in cross-disciplinary thematic learning under three-dimensional support.

The essential support conditions, specific activities, and positive impacts brought about by the implementation of cross-disciplinary thematic learning are closely interconnected and collectively form the logical framework for formulating the research questions. This logical framework serves to link together the specific research content, as illustrated in Figure 2.

As shown in Figure 2, this study proposes three dimensions based on curriculum theory: student foundation, disciplinary resources, and social environment. Within the framework of these three dimensions, cross-disciplinary thematic learning activities occur, which include understanding and representation, planning and execution, evaluation, and reflection. These cross-disciplinary thematic activities are part of the problem-solving process and a means for students to engage in, expand, and develop their problem-solving skills.

## 3 Methodology

### 3.1 Research methods and participants

An optimal methodological approach for addressing research inquiries on cross-disciplinary thematic learning impacts and their influence on students' problem-solving abilities would be to investigate the process in a real-world environment (Yin, 2009). Specifically, to study multiple case studies, over time, to understand behavior and why it occurs. In this study, observation and interview methodologies were integrated to ascertain the actual behaviors of students in resolving cross-disciplinary thematic tasks, along with the underlying reasons behind their actions. In combination with research questions, this study mainly starts from three aspects in terms of the selection of schools and objects. First, choose schools that are easy to communicate, encourage and support academic research, and have the necessary conditions and space for operable empirical research. Second, the selection of research participants mainly refers to the situation of the most recent exam, that is, the overall level of the participants in the whole grade, and select students whose entire level is at the upper level of the grade. Third, the sampling of primary school students participating in the test mainly considers the exclusion of some irrelevant interference factors, such as normal intellectual level, no mental illness or physical disorders, and does not include students who have received special training related to the development of the ability to discover problems, so as to minimize the interference caused by special circumstances in the estimation of empirical research results. Based on this, 47 study participants with M Middle School ranked at the upper and upper level in grade 9 were included in the empirical study scope, including 28 boys with an average age of 14 years and 19 girls.

### 3.2 Research procedure

Given the supporting conditions required for cross-disciplinary thematic learning, a total of five cross-disciplinary thematic learning tasks that meet the criteria from the Mathematics and History curriculum standards (2022 edition), as shown in Table 1, were selected. The purpose of this study is to explore the impact of cross-disciplinary thematic learning on students' problem-solving abilities which necessitated mitigating the potential influence of confounding variables on the reliability and validity of the research results (Johnson and Chistensen, 2015). Therefore, one cross-disciplinary thematic learning practice activity per week was conducted following a sequence of Task 1 to Task 5.

**Table 1:** Overview of cross-disciplinary thematic learning tasks.

**Tasks**	**Theme**	**Task drive**	**Expected outcomes**	**Interdisciplinary**
Task 1	Development of land and water transportation in history	Collecting and organizing historical materials, synthesizing interdisciplinary knowledge, understanding the development of land and water transportation in different historical periods, and recognizing the significant role of land and water transportation development.	Research report on the role of land and water transportation construction in national governance, economic exchange, social life, etc.	History, Geography, Ethics and Rule of Law, Science.
Task 2	Drawing campus maps	Conducting field measurements, integrating knowledge of plane geometry and its spatial relationships, sketching the three elements of maps, and acquiring relevant art knowledge to draw maps.	Campus floor plan map.	Mathematics, Geography, Fine Arts.
Task 3	Discovering history around us	Activities such as collecting items, arranging data, compiling and framing, and gaining insights into historical changes and people's lives.	Compilation of the *Exploring History Around Us* picture album.	History, Geography, Ethics and Rule of Law, Language Arts, Arts.
Task 4	Sports activities and heart rate	Exploratory activities related to sports health, and safety.	Research report on the relationship between sports activities and heart rate.	Physical Education, Mathematics, Biology.
Task 5	Nutritious lunch	Investigating, calculating, and analyzing various nutrients in lunch, designing a nutritious lunch menu.	Nutritious lunch recipes.	Nutrition, Mathematics, Science.

**Figure 3:**
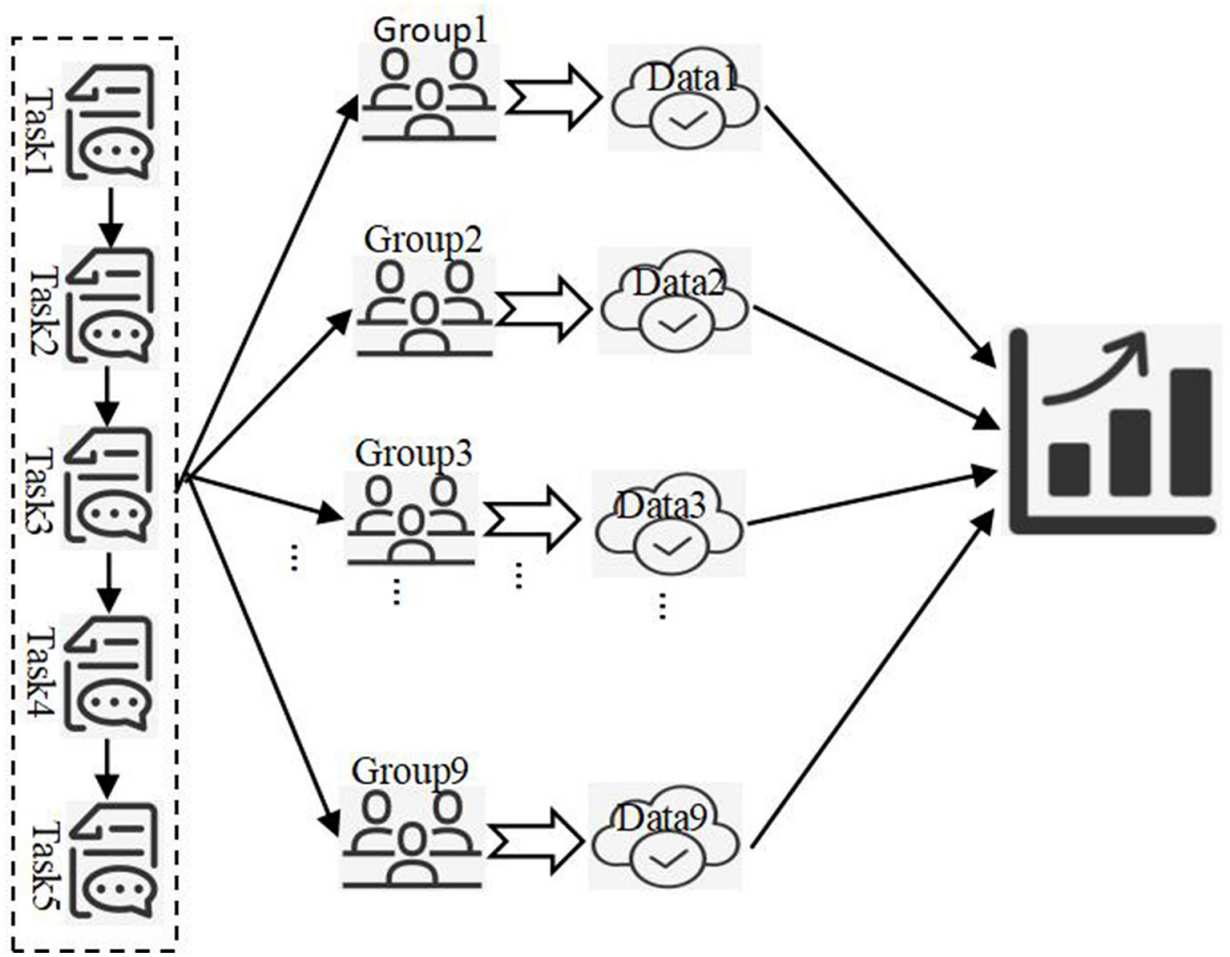
Research framework for developing students' problem-solving skills through interdisciplinary theme-based learning.

The class of 47 students was divided into nine groups, each group consisting of 5–6 students. Students in each group were encouraged to collaborate autonomously, to facilitate completing problem-solving tasks systematically, as per cross-disciplinary thematic learning. Qualitative data collected during the completion of these tasks were used to assess the level of students' problem-solving abilities. The average levels of problem-solving abilities among the nine groups of students as they completed each task were plotted on line charts and bar graphs. The changes and trends in their problem-solving abilities were noted as the frequency of their participation in cross-disciplinary thematic practice activities increased. Based on the process logic of developing problem-solving abilities through cross-disciplinary thematic learning, the researchers of this study hypothesized that adequate support in student foundation, disciplinary resources, and social environment would enhance students' problem-solving abilities. Therefore, the rationality of this theoretical study was further analyzed by incorporating interview data. The research flowchart based on the study's approach to developing students' problem-solving abilities through cross-disciplinary thematic learning is illustrated in Figure 3.

### 3.3 Research measures

#### 3.3.1 Analysis framework for problem-solving abilities

To better align with problem-solving scenarios facilitated by cross-disciplinary thematic learning, the study researcher/s adapted and refined the problem-solving cognitive processes outlined by Gick (1986) and OECD (2017) and proposed a definition for student problem-solving abilities. Student problem-solving abilities refer to the capacity of students to comprehensively integrate knowledge from various disciplinary domains, accurately comprehend problems by establishing mental representations, formulate solutions to problems based on specific contexts and conditions, adjust these solutions in complex environments, and evaluate and reflect on the problem-solving process. Based on this definition, student problem-solving abilities can be analyzed and assessed through three cognitive processes: understanding and representation, planning and execution, and evaluation and reflection. Detailed explanations and observational indicators of each cognitive process are provided in Table 2. Based on the potential performances of students in the three cognitive processes—understanding and representation, planning and execution, and evaluation and reflection—the researcher/s formulated specific performances for student problem-solving ability Levels 0 to 3, using combinatorics from mathematics. These levels are represented as follows: Level 0 is denoted by $C_{3}^{0}$, Level 1 by $C_{3}^{1}$, Level 2 by, and Level 3 by $C_{3}^{3}$. For example, the three observed indicators corresponding to the understanding and representation of cognitive processes are as follows: ①analyzing key information in task situations; ②posing appropriate questions; ③forming a series of sub-problems conducive to problem-solving.$C_{3}^{0}$ (Level 0) indicates that students can achieve 0 of the three observed indicators; $C_{3}^{1}$ (Level 1) indicates that students can achieve any one of the three observed indicators; (Level 2) indicates that students can achieve any two of the three observed indicators; $C_{3}^{3}$ (Level 3) indicates that students can achieve all three of the observed indicators. Similarly, the planning and execution, evaluation and reflection cognitive processes, each corresponding to three observed indicators, are denoted by $C_{3}^{i}$ (*i* = 0,…,3), representing any *i* of the three observed indicators, to indicate the specific performance of students at Levels 0–3.

**Table 2:** Observation indicators of problem-solving ability.

**Cognitive processes**	**Connotation**	**Observation indicators**
Understanding and Representation	Understanding the meaning of information in the task context, forming an appropriate problem space.	① Analyzing and selecting key information in the task context; ② Formulating appropriate questions; ③ Generating a series of sub-problems conducive to problem-solving.
Planning and Execution	Formulating solutions for problems based on specific contextual conditions and existing disciplinary knowledge and skills. Executing, monitoring, or adjusting the problem-solving solutions in complex environments.	① Selecting and applying contextual information and disciplinary knowledge and skills that can solve the problem; ② Approaching from multiple perspectives to generate multiple preliminary solutions that can effectively solve the specific situational problem and selecting a solution that is both practical and effective; ③ Adjusting or refining the problem-solving solution in response to feedback information during the execution of the solution.
Evaluation and Reflection	Evaluating, summarizing, and reflecting on the problem-solving process.	① Independently seeking criteria for evaluating the results of the problem; ② Reflecting on and optimizing different perspectives such as the accuracy of the problem representation, the feasibility of the solution, and the effectiveness of the results; ③ Appreciating the gains from the problem-solving process.

#### 3.3.2 Interview outline

In this study, the researcher/s collected qualitative data from students using interviews during the task completion. Specifically, the interviews were used to assess students' problem-solving ability level and to understand how their basic support, disciplinary resources, and social environment support contributed to enhancing their problem-solving abilities. Therefore, the interview outline consists of two main parts, detailed in the Appendix. To be more specific, the interview outline of the first part is mainly aimed at collecting numerical values used to express students' problem-solving ability level. By having conversations with students, we judge whether students have met the requirements of observation indicators for problem-solving ability mentioned in Table 2 and combined with the students' interview content to determine whether students' cognitive process of solving different problems and meet the requirements of observation indicators. Then, with the help of the idea of combining numbers in mathematics, the interview content is transformed into scores that can represent students' problem-solving abilities more intuitively and clearly, such as 0 points, 1 points, 2 points, and 3 points, to obtain students' problem-solving abilities. The interview outline of another part aims to deeply explore the supporting elements that determine interdisciplinary theme learning tasks that can improve students' problem-solving ability. Based on the interview outline of this part, we have a dialogue with students. After sorting and analyzing the interview data, the original text data in the interview are presented in the “discussion” stage to retain the original text during the interview process, and more vividly illustrate the three-dimensional support for the development of students' problem-solving ability through interdisciplinary theme learning. During the interviews, the researcher/s employed the stimulated recall method. Specifically, students were stimulated to recall their thoughts during the interdisciplinary task-solving process by revisiting their completed work and explaining the reasons behind their choices at that time (Huang, 2014).

### 3.4 Reliability and validity of the study

To ensure the reliability of the “Problem-Solving Ability Level Analysis Framework” tool, three experts were invited to evaluate and score the observation indicators of “problem-solving ability” according to their appropriateness. The scoring criteria for appropriateness were rated as “not appropriate,” “appropriate after modification,” and “appropriate,” corresponding to scores of 1–3, respectively. According to expert judgments, the mean scores for the appropriateness of all indicators in this study were above 2.7, indicating that these indicators adequately reflected the traits intended to be measured in this study (Yu et al., 2022). Furthermore, to enhance the credibility and objectivity of the scoring results, the study examined the consistency of the level assignments by the three assessors. Correlation analysis of the three assessors' ratings of problem-solving ability levels for the five cases yielded consistency coefficients of 0.882, meeting the requirements for inter-rater reliability (Wu, 2010).

The researcher/s employed methodological and data triangulation to enhance validity. Specifically, they initially used observation methods to observe students' actual behaviors during task completion, followed by focus group interviews to delve deeper into the reasons behind these behaviors. Additionally, to mitigate potential researcher biases in selective observation and data recording, researcher triangulation was used, involving multiple researchers in data collection, analysis, and interpretation.

## 4 Discussion

### 4.1 The development of students' problem-solving abilities facilitated by interdisciplinary theme-based learning

This study combines observational and interview data based on the Interdisciplinary Problem-Solving Ability Level Analysis Framework. The researcher/s assigned levels to students' understanding and representation (P1), planning and execution (P2), and evaluation and reflection (P3) processes during the completion of five interdisciplinary theme-based learning tasks by nine groups of students. Specific results are detailed in Table 3.

**Table 3:** Average problem-solving ability levels.

**Task**	**P1**	**P2**	**P3**	**P**
Task 1	1.56	1.56	1.45	1.52
Task 2	2.45	2.34	1.56	2.12
Task 3	2.56	2.45	1.56	2.19
Task 4	2.45	2.56	2.56	2.52
Task 5	2.89	2.78	2.45	2.71

**Figure 4:**
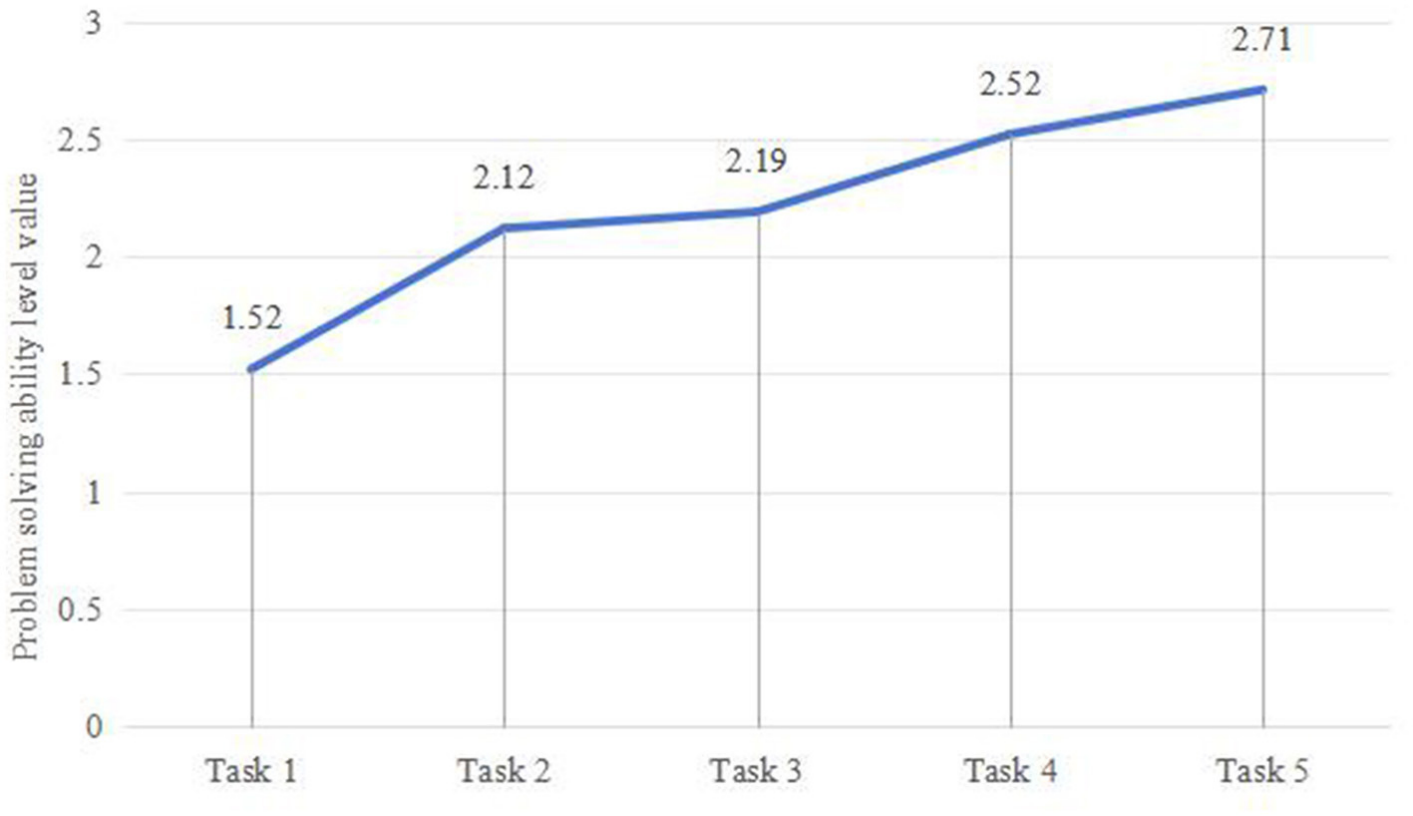
Changes in problem-solving ability levels.

**Figure 5:**
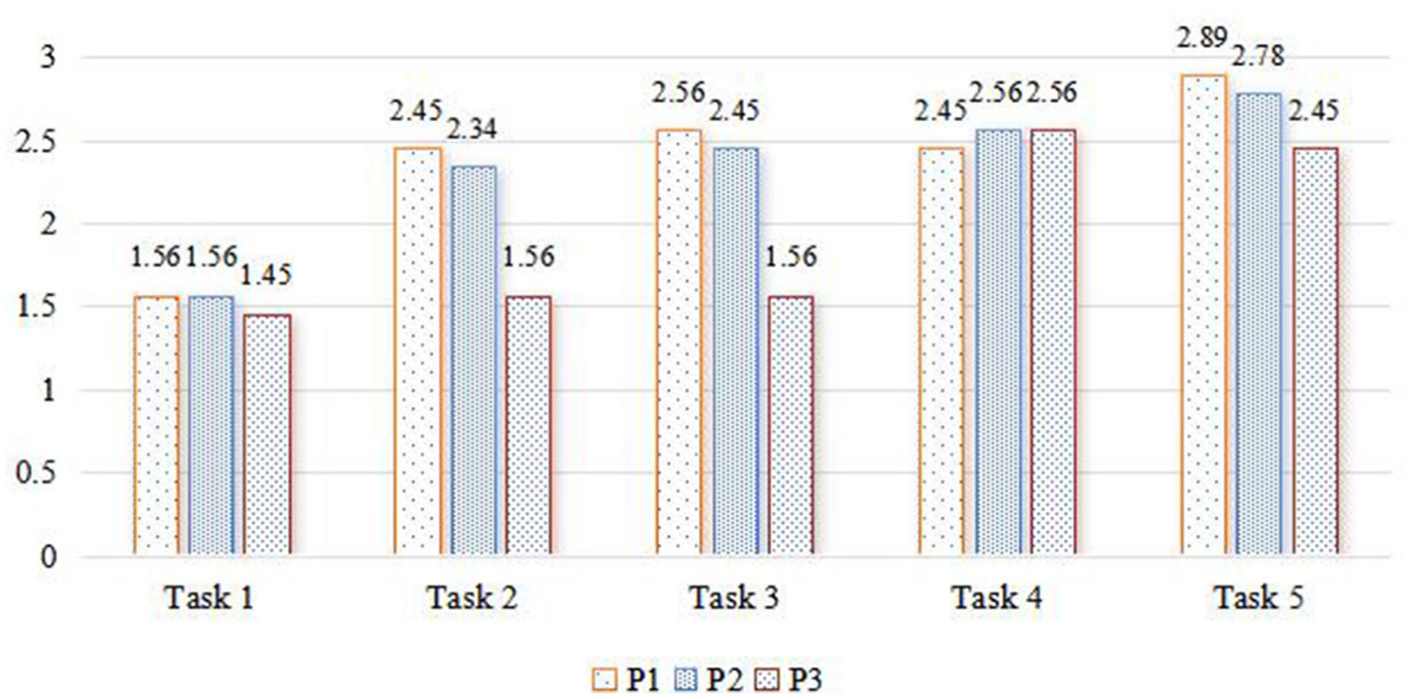
Changes in P1–P3 levels.

Comparing the mean values of problem-solving abilities (P) presented in Table 3, it can be seen that students' problem-solving abilities improved as the interdisciplinary theme-based learning activities (Task 1 to Task 5) progressed (Figure 4). Among the three processes—understanding and representation, planning and execution, and evaluation and reflection, only P2 showed a gradual upward trend, indicating that the growth trend of visualized planning and execution abilities was more apparent. However, P1 and P3 did not exhibit a consistent incremental trend. Specifically, P1 showed a fluctuating trend between Task 1 and Task 5—initially increasing, then decreasing, and then increasing again. This suggests that the performance level of understanding and representation thinking processes is related to the type and difficulty of tasks. While P3 showed a stable trend between Task 2 and Task 3, overall it continued to increase, indicating that the development of reflection and evaluation, as higher-order thinking processes, was slower and required prolonged penetration and guidance. Detailed information is provided in Figure 5.

To better understand the specific development of students' problem-solving abilities during the completion of interdisciplinary theme-based learning tasks, the researcher/s selected three representative groups from the five tasks. They then provided a detailed overview of the current level of problem-solving abilities during the completion of interdisciplinary tasks, as shown in Table 4.

**Table 4:** Changes in problem-solving ability levels for three groups of students.

**Task**	**Problem solving ability structure category**	**Group 1**	**Group 2**	**Group 3**
Task 1 → Task 2	P1	0 → 1	1 → 1	2 → 2
	P2	1 → 1	1 → 1	2 → 2
	P3	0 → 0	1 → 1	2 → 2
Task 2 → Task 3	P1	1 → 3	1 → 2	2 → 3
	P2	1 → 2	1 → 2	2 → 2
	P3	0 → 1	1 → 2	2 → 2
Task 3 → Task 4	P1	3 → 1	2 → 2	3 → 3
	P2	2 → 2	2 → 2	2 → 3
	P3	1 → 2	2 → 2	2 → 2
Task 4 → Task 5	P1	1 → 3	2 → 3	3 → 3
	P2	2 → 2	2 → 2	3 → 3
	P3	2 → 2	2 → 3	2 → 3

Combined with the changes in the P1 scores of Group 1 students, in the first round, from Task 1 to Task 2, students' P1, P2, and P3 scores were generally low, with little variation. Moving into the second round from Task 2 to Task 3, students' P1, P2, and P3 scores generally increased, with significant growth observed in the P1 aspect. However, in the third round from Task 3 to Task 4, students' P1 scores showed a significant decline. This indicates that interdisciplinary theme-based learning, with history and mathematics subjects as “anchors,” may be influenced by the characteristics of these “anchor” subjects, resulting in fluctuations in students' problem-solving abilities. In contrast, the changes in P1 scores for Groups 2 and 3 students were not as significant as those for Group 1 students. Their scores remained unchanged or steadily increased. This suggests that interdisciplinary theme-based learning not only receives support from history and mathematics subjects but also benefits from other supportive conditions. This mitigates the adverse effects of subject characteristics on students' problem-solving abilities leading to a more balanced development of their problem-solving abilities.

In summary, it is necessary to continuously carry out interdisciplinary thematic learning activities that can attract students to actively participate in the above-disciplinary thematic learning activities, so as to strengthen the close connection between interdisciplinary thematic learning and the development of students' problem-solving ability, thereby effectively promoting the development of students' problem-solving ability (Guo et al., 2025). Specifically, by extending the process of cross-disciplinary theme learning activities, it helps to coordinate the development rhythm and change trend of the cognitive processes of problem-solving ability P1, P2, and P3, thereby increasing the possibility that P1, P2, and P3 can jointly help students improve their overall problem-solving ability. It can be seen that through observation method, this study obtains specific score data on students' problem-solving ability performance during the interdisciplinary theme learning task. The changes in score data can help us intuitively express the development brought by interdisciplinary theme learning to students' problem-solving ability, and visualize relatively implicit and potential abilities in response to the first research question raised in this study.

However, students' problem-solving ability means a complex comprehensive ability (Gu et al., 2022). It is difficult to interpret students' problem-solving ability based solely on scores, and use the level analysis framework of problem-solving ability as a research tool. The indicators listed in it are difficult to fully include students' dynamic changes in real situations. To a certain extent, there is the possibility of limiting students' uncertainty in real situations, weakening students' problem-solving ability development vitality, and narrowing the development space for students' problem-solving ability. Therefore, it is necessary to use observation method to obtain the score data for students' problem-solving ability to characterize students' problem-solving ability, supplemented by methods such as flexible and profound characteristics, so as to highlight respect for uncertainty in the real situation and the observation of personalized performances presented by students with different life courses.

### 4.2 Interdisciplinary theme-based learning and three-dimensional support

Through further analyzing the interview content, it can be seen that the interdisciplinary theme learning task includes three-dimensional support composed of students' foundation, subject resources, and social environment to achieve the development of interdisciplinary theme learning and create an open, inclusive and authentic atmosphere for students' problem-solving ability development (Xu, 2024).

#### 4.2.1 Student foundation support for the development of problem-solving abilities

Student foundation support refers to the knowledge, skills, processes, methods, emotions, and values students have acquired in their learning experiences across various disciplines. It can be regarded as a raw reserve for students to solve real problems close to real life to support students to integrate and apply them flexibly in interdisciplinary themed learning activities (Xu et al., 2015). This support lays a solid foundation for students to integrate and apply their learning in activities, thus enabling them to utilize their original reserves to solve problems flexibly. Student foundation support mainly manifests as the basic content students already possess and the new content they acquire when participating in interdisciplinary theme-based learning tasks. Both types of content provide strong support for students to solve problems in interdisciplinary theme-based learning tasks.

As per the interview data, students' responses emphasized the existing basic content from various disciplines. One student's response is as follows:

“*Regarding the historical development of land and water transportation, we recalled the content we learned in seventh grade about the opening of the Silk Road, as well as the geographical locations of Zhuo County and Yu Hang that we studied in geography. We utilized our map-reading skills to understand how they were connected and where these two ancient place names are located today. Additionally, we applied the skills of summarizing and synthesizing texts that we acquired in Chinese language arts*.”

In the process of participating in interdisciplinary thematic learning tasks and considering the need to solve specific problems, students mentioned evidence of newly acquired foundational content from various subjects. Another student's response is as follows:

“*When participating in the task of sports activities and heart rate, we did not have the relevant sports knowledge matching this task, such as what heart rate range is healthy during exercise. Therefore, we proactively learned about this knowledge, which helped us better understand the relationship between types of exercise, exercise time, and heart rate*.”

#### 4.2.2 Subject resource support for developing students' problem-solving abilities

Subject resource support refers to the resources within the school that have characteristics of various subjects, providing rich reserves for interdisciplinary thematic learning. It facilitates students' problem-solving cognition and practice through multiple pathways (Garry et al., 2020). As per the interview data, students mentioned obtaining appropriate assistance matching specific interdisciplinary tasks from different subjects within the school. Students' responses are as follows:

“*In drawing a campus map, we consulted the geography teacher for knowledge about scale, orientation, and legend. In art class, we borrowed drafting paper and paint, and learned how to draw irregular shapes*,” and…

“*When participating in the task of discovering history around us, we searched for historical materials about the Han, Ming, and Qing dynasties in the library. We utilized the network resources provided in information technology class to collect images of clothing from the Han, Ming, and Qing dynasties. Additionally, we used the drawing skills learned in art class to illustrate the clothing for presentation*.”

#### 4.2.3 Social environment support for developing students' problem-solving abilities

Social environment support refers to the real problems and practical situations originating from social life (D'Zurilla and Goldfried, 1971). Real problems run through the entire process of interdisciplinary thematic learning tasks bridging the gap between students and interdisciplinary thematic learning tasks and enhancing students' interest in problem-solving. Practical situations create authentic contexts for interdisciplinary thematic learning tasks, making it easier for students to gain embodied experiences in problem-solving (Techakosit and Nilsook, 2018; Top and Sahin, 2015; Bennett and Monahan, 2013). As per the interview data, students mentioned real problems and practical situations. Some examples are as follows:

“*The task of solving the relationship between physical exercise and heart rate, it's something we encounter in our daily lives, and because we enjoy physical education classes, we are very interested in solving this problem. It not only increases our knowledge of how to exercise healthily but also provides helpful advice for how to effectively relieve ‘screen time syndrome'*.”

“*The development of water and land transportation is closely related to our daily social life. Some students in our class come from Shandong Province, and they travel to Sichuan for school by plane. This provided us with a breakthrough in solving this problem*.”

In conclusion, students' foundations, subject resources, and social environment play their respective unique roles in the implementation and effectiveness of interdisciplinary thematic learning, collectively constituting the necessary support for students to develop their problem-solving abilities (Phelan, 2011). In interdisciplinary thematic learning tasks, students' foundations provide them with the original reserves for problem-solving (Ma, 2021), subject resources offer students multiple pathways for problem-solving (Herschbach, 2011), and the social environment enables students to gain embodied experiences in problem-solving (Falkenberg, 2015). Therefore, interdisciplinary thematic learning with the support of these three dimensions can facilitate and gradually develop students' problem-solving abilities. Under the guidance of existing theories, this study found that the curriculum is closely related to the three elements of students, subjects and society, in order to further uncover the relationship between interdisciplinary theme learning activities and the three elements, as well as its role in the development of students' problem-solving ability. Therefore, with the help of the interview method, the smooth progress of interdisciplinary thematic learning activities is revealed, which contains support from three dimensions of students, subjects and society, so that deep influencing factors that improve students' personal problem-solving ability can emerge in response to the second research question raised in this study.

However, the application of interview method in research is easy to carry guidance related to research needs and the researcher's personal main views. This has the risk of interfering with students' real experience when participating in interdisciplinary theme learning tasks to a certain extent, and the phenomenon that students weaken or conceal their personal subjective judgments to cater to the interviewer's questions will lead to the persuasiveness of using interview materials as evidence to extract interdisciplinary theme learning to affect the development of students' problem-solving ability, and at the same time, the accuracy of positioning of three influencing factors, including students, disciplines and society, in the interdisciplinary theme learning tasks. Therefore, more reasonable research methods need to be further supplemented, and bridged with the interview method, deeply revealing the specific operating principles of interdisciplinary theme learning to develop students' problem-solving abilities, so as to improve the possibility of transfer to similar learning situations.

## 5 Conclusion

**Figure 6:**
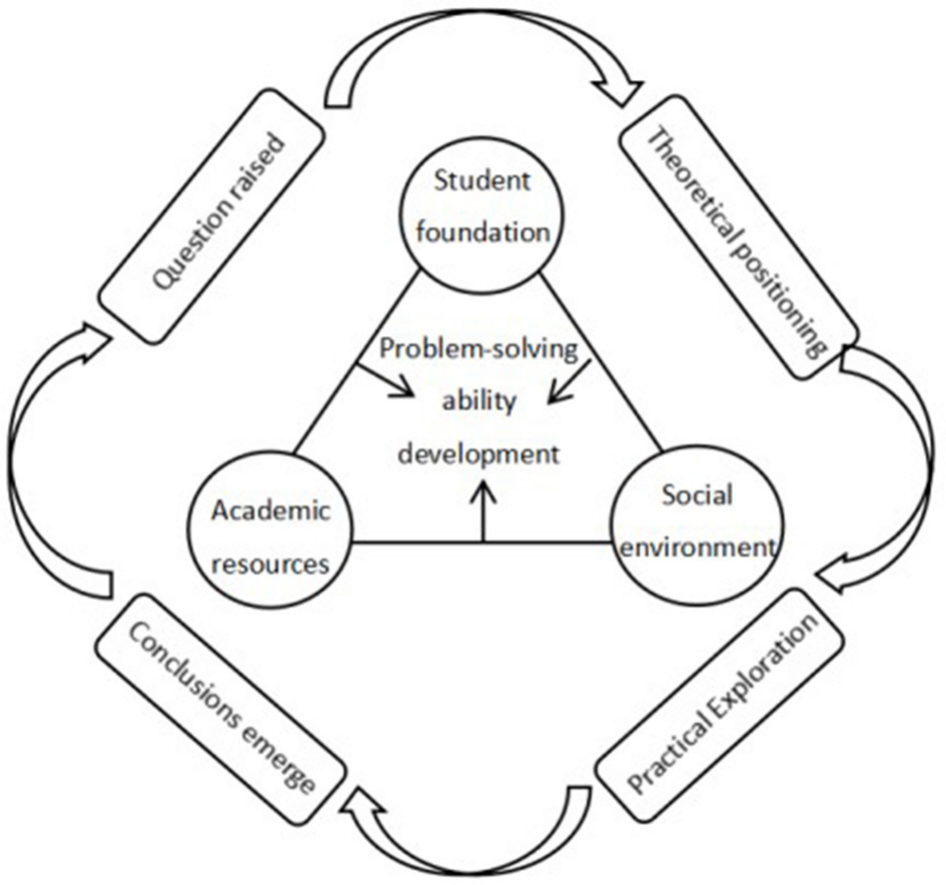
Summary of research process and conclusions.

Students' problem-solving capabilities evolve through engagement in five distinct interdisciplinary thematic learning tasks. This underscores the potential of interdisciplinary thematic learning as a pivotal conduit for nurturing students' interdisciplinary problem-solving prowess and fostering its growth. Furthermore, an analysis of students' interview data shows that interdisciplinary thematic learning is facilitated by the collective support of students' foundations, subject resources, and societal milieu, catalyzing their problem-solving acumen. Hence, initiating interdisciplinary thematic learning from the vantage points of students, subjects, and society can better play a role in developing students' problem-solving abilities. In order to present the research conclusions of this study clearly and concisely, additional explanations are performed by drawing, as shown in Figure 6.

## 6 Limitations and prospects

In terms of the selection of research subjects, this study employed a multiple-case design that concurrently investigated the problem-solving abilities of nine student groups during interdisciplinary thematic learning to bolster the credibility of research outcomes by studying multiple cases. However, it's imperative to acknowledge that the breadth of analysis derived from multiple case studies often comes at the expense of research depth (Yin, 2009), in the form of single-case quasi-experimental design studies. Future research on this subject should continue to harness the strengths of multiple case studies while concurrently undertaking single-case, quasi-experimental design studies to augment research depth, thus enabling the formulation of more robust and comprehensive conclusions and recommendations. In addition, in terms of research perspective, this study mainly starts from the perspective of students' independent learning, and explores the improvement of problem-solving ability of students in the process of participating in interdisciplinary theme learning tasks. For the development of students' problem-solving abilities, this is obviously not enough. Therefore, when choosing a research perspective in the future, it is necessary to incorporate considerations of teacher guidance (Lin et al., 2023), digital technology (Majid et al., 2024), and computing thinking skills (Tripon, 2022).

## Data Availability

The original contributions presented in the study are included in the article/supplementary material, further inquiries can be directed to the corresponding author.

## Appendix

1. Interview guide for problem-solving skills
1.1 Interview Guide for understanding and representation of the cognitive process
(1) Did you analyze the background information of the task context? What information did you analyze?
(2) Did you understand the objectives of this task? What were they?
(3) Did you formulate a series of sub-problems conducive to problem-solving? What were they?
1.2 Interview Guide for planning and execution cognitive process
(4) Which contextual information or interdisciplinary knowledge and skills did you select for problem-solving?
(5) What possible solutions did you design for problem-solving? When executing, which pre-selected solutions did you choose? Why?
(6) What difficulties did you encounter during the execution process? How did you deal with them?
1.3 Interview Guide for evaluation and reflection cognitive process
(7) Did you search for criteria to evaluate the problem's outcome? What were they? Why?
(8) Do you wish to optimize the problem-solving process? Specifically, where do you want to optimize? Why?
(9) What achievements did you gain during the problem-solving process? Why?
2. Interdisciplinary theme learning task support system interview outline
(1) Do you possess the interdisciplinary knowledge and skills required to solve this task? If there are any deficiencies, what would you do? Why?
(2) What kind of support from different disciplines do you want to receive during the process of solving this task?
(3) Can real-world problems and practical scenarios derived from social life motivate you to solve problems better?
